# Autochthonous Human Case of Seoul Virus Infection, the Netherlands

**DOI:** 10.3201/eid2412.180229

**Published:** 2018-12

**Authors:** Caroline Swanink, Johan Reimerink, Jet Gisolf, Ankje de Vries, Mark Claassen, Liesbeth Martens, Toos Waegemaekers, Harry Rozendaal, Stasja Valkenburgh, Tabitha Hoornweg, Miriam Maas

**Affiliations:** Rijnstate Hospital, Arnhem, the Netherlands (C. Swanink, J. Gisolf, M. Claassen);; National Institute for Public Health and the Environment, Bilthoven, the Netherlands (J. Reimerink, A. de Vries, T. Hoornweg, M. Maas);; Municipal Health Service Gelderland-Midden, Arnhem (L. Martens, T. Waegemaekers);; Dutch Food and Consumer Products Safety Authority, Utrecht, the Netherlands (H. Rozendaal, S. Valkenburgh)

**Keywords:** Seoul virus, orthohantavirus, SEOV, hemorrhagic fever with renal syndrome, HFRS, rats, source investigation, zoonotic infection, viruses, the Netherlands, zoonoses

## Abstract

Orthohantaviruses are a group of rodentborne viruses with a worldwide distribution. The orthohantavirus Seoul virus (SEOV) can cause hemorrhagic fever with renal syndrome in humans and is distributed worldwide, like its reservoir host, the rat. Cases of SEOV in wild and pet rats have been described in several countries, and human cases have been reported in the United Kingdom, France, Canada, and the United States. In the Netherlands, SEOV has previously been found in wild brown rats. We describe an autochthonous human case of SEOV infection in the Netherlands. This patient had nonspecific clinical symptoms of an orthohantavirus infection (gastrointestinal symptoms and distinct elevation of liver enzymes). Subsequent source investigation revealed 2 potential sources, the patient’s feeder rats and a feeder rat farm. At both sources, a high prevalence of SEOV was found in the rats. The virus closely resembled the Cherwell and Turckheim SEOV strains that were previously found in Europe.

Orthohantaviruses are a family of rodentborne viruses with a worldwide distribution. Human infection can occur when virus-contaminated aerosols of rodent excreta are inhaled while entering or cleaning rodent-infested areas (*1*). Infection can also be transmitted by rodent bites or when orthohantavirus contaminated materials are directly introduced into broken skin or conjunctiva. The clinical syndromes that are associated with severe disease are hemorrhagic fever with renal syndrome (HFRS) and orthohantavirus cardiopulmonary syndrome (HCPS). HFRS cases are found in large parts of Europe and Asia, whereas HCPS cases are found in North America and South America (*2*). The orthohantavirus Seoul virus (SEOV) causes HFRS of medium severity and was originally found in Asia. In Europe, cases of SEOV in wild and pet rats have been described in the United Kingdom, Belgium, Sweden, and France (*3*–*6*). Additional outbreaks have been reported in Canada and the United States (*7*). The first non–laboratory-related human infections with SEOV in Europe were reported in the United Kingdom and France in 2012, although retrospectively earlier cases might have occurred (*8*–*10*). From 2013 on, multiple additional human cases were reported in the United Kingdom and France (*3*,*11*,*12*). In the United States, domestic cases of HFRS attributable to SEOV have been described since 1994 (*13*). In the Netherlands, SEOV has been reported in wild rats (*14*,*15*). In this article, we describe an autochthonous human case of SEOV infection in the Netherlands and the subsequent source investigation.

## Case Description

In September 2016, a 28-year-old man sought medical care at Rijnstate Hospital (Arnhem, the Netherlands); he reported having fever, vomiting, abdominal cramps, and diarrhea of 7 days’ duration. He had a history of gamma-hydroxubutyrate (GHB) addiction and tobacco use (10 cigarettes/d). He denied drinking alcohol. He had no history of travel. The patient mentioned that he had been bitten regularly while handling live rats at his work at a rat breeding farm and by live rats that he kept for his reptiles at home. He also mentioned he had been swimming in the Rhine River, in which rats can be found. Physical examination revealed a sweating, obese, ill patient with normal blood pressure, tachycardia (113 beats/min), and a temperature of 38.6°C. No abnormalities on auscultation of heart and lungs or lymphadenopathy were found. Abdominal examination revealed a painful enlarged liver. No abnormalities of the skin were recorded. We have summarized the patient’s laboratory test results (Table 1). Ultrasound revealed normal aspect of liver and gall bladder and a slightly enlarged spleen (17.2 cm). The patient was hospitalized with an initial diagnosis of gastroenteritis, colitis, or leptospirosis. Antibiotic treatment with cefuroxime, metronidazole, and doxycycline was started. Blood cultures remained negative. A serum sample from the patient, taken at admission, tested negative for hepatitis A, B C, and E viruses; HIV; *Treponema pallidum* (syphilis); cytomegalovirus; Epstein-Barr virus; and *Leptospira* spp. The patient did not have signs of acute kidney injury and showed only a mild proteinuria of 0.25 g/L in a single urine sample. He was tested for orthohantavirus infection because he mentioned that he was bitten by rats regularly. The father of the patient, who took care of the reptiles occasionally, and the patient’s partner, who did not have any contact with the reptiles, were not feeling ill.

**Table 1:** Laboratory test results for a patient diagnosed with Seoul virus infection, the Netherlands, September 2016*

Laboratory test	Reference range	Day 1†	Day 3‡
C-reactive protein, mg/L	<10	32	59
Leukocytes, 10^9^ cells/L	4.0–11.0	5.0	12.3
Lymphocytes, 10^9^ cells/L	1.0–3.5	NT	8.27
Atypical lymphocytes	–	NT	+
Platelets, 10^9^ cells/L	150–400	72	79
Creatinine, µmol/L	60–110	78	72
Alanine aminotransferase, U/L	<45	114	211
Aspartate aminotransferase, U/L	<35	123	283
Lactate dehydrogenase, U/L	<250	753	1906
Bilirubin, µmol/L	<17	12	10
Creatinine kinase, U/L	<170	NT	677

*NT, not tested; –, negative; +, positive. †Day 1 of hospitalization, 7 days after onset of symptoms. ‡Day 3 of hospitalization, 9 days after onset of symptoms.

## Material and Methods

### Human SEOV Diagnostics

For detection of hantavirus IgG and IgM, we used an immunofluorescent assay (IFA) with mosaic slides containing SEOV and other orthohantaviruses Puumala virus, Sin Nombre virus, Hantaan virus, Dobrava-Belgrade virus, and Saaremaa virus (Euroimmun, Lübeck, Germany), according to the manufacturer’s instructions (*16*). The titer was defined as the last sample dilution for which the fluorescence was identifiable, and a titer >1:32 was considered positive. Serum samples of the patient and 2 close contacts were tested for hantavirus antibodies. Total nucleic acid was extracted from patient serum by using the MagNAPure 96 system (Roche, Basel, Switzerland) and tested for SEOV RNA by using a hantavirus genus–specific real-time reverse transcription PCR (rRT-PCR), as described by Kramski et al. (*17*).

### Investigation of Feeder Rats Owned by the Patient

Because the patient kept feeder rats at home and these rats are a known source of SEOV infection, the rats were collected for source investigation. At the time of investigation, the patient had 5 live and 5 frozen feeder rats at home. The rats were housed in a domestic residence and were 7–13 months old. All likely originated from a feeder rat breeding farm, where the patient worked regularly as a volunteer, although the patient gave contradicting information about this. All available rats were tested for SEOV virus. The 5 live rats were euthanized, and serum and lung tissues were collected. For the frozen rats, serum was collected by vortexing and centrifugation of the rat hearts, as described previously (*15*). Antibodies in rat serum were detected by using a human SEOV ELISA (Hantavirus Dobrava/Hantaan IgG Elisa; Progen Biotechnik GmbH, Heidelberg, Germany), which was adapted to enable detection of IgG in rats. Rabbit-α-rat horseradish peroxidase-labeled IgG (Sigma-Aldrich Chemie B.V., Zwijndrecht, the Netherlands) was used as conjugate at a 1:5,000 dilution. A cutoff value was based on the average OD of negative control rat serum + 3 × SD (in this case, a value of 0.2–0.3).

**Figure 1:**
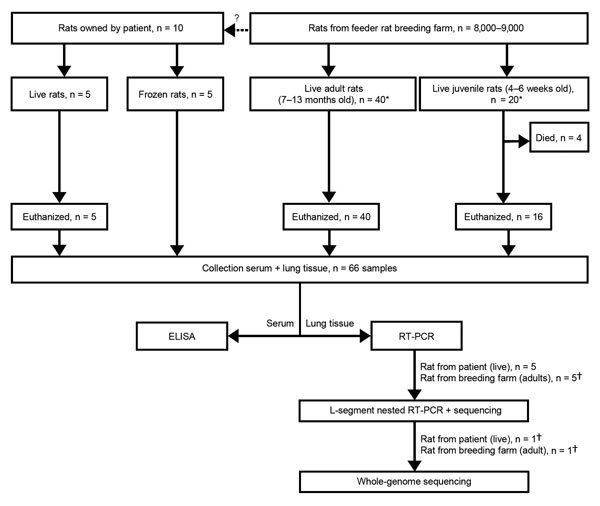
Flowchart depicting the selection and subsequent testing of feeder rats in a source investigation following detection of a human case of Seoul virus infection, the Netherlands, September, 2016. *Rats were randomly picked; †rats were randomly picked from among Seoul virus–positive animals. RT-PCR, reverse transcription PCR.

For euthanized and frozen rats, lung tissue was collected in RNAlater (Applied Biosystems, Foster City, CA, USA) and stored at −80°C. Lung tissue was disrupted in MagNA Pure 96 External Lysis Buffer (Roche) by using Lysis matrix D (MP Biomedicals, Santa Ana, CA, USA) and Fast Prep FP120 homogenizer (Thermo Savant, Carlsbad, CA, USA). Total nucleic acid was isolated by automated nucleic acid extraction by using the MagNA Pure 96 system (Roche). As a first screening, a hantavirus genus–specific rRT-PCR was performed on lung tissue, as described previously. Subsequently, a selection of the samples was confirmed with a nested rRT-PCR assay of the large (L) segment, as described by Klempa et al. (*18*). The resulting fragments were purified with ExoSAP-IT PCR clean-up (Isogen Life Science, Utrecht, the Netherlands) and sequenced by Baseclear (Leiden, the Netherlands). For clarity, details of the selection of rats and the subsequent experimental procedures are summarized in Figure 1.

### Investigation of Rats from the Feeder Rat Breeding Farm

The feeder rats from the patient’s home likely originated from the feeder rat breeding farm where the patient volunteered. At the time of the investigation, the feeder rat breeding farm housed 8,000–9,000 rats, 7 rabbits, and approximately 30 gerbils, 100 mice, and 60 snakes (constrictors). Adult rats at the farm were housed in 40 open boxes that measured 1 m × 1 m. About 30 rats were kept per box, of which ≈5–10 were male and 20–25 were female. Smaller boxes were available for pregnant female rats, female rats with pups, and juvenile rats.

Sixty rats, of which 40 were adults (age 7–13 months) and 20 were juveniles (age 4–6 weeks), were collected. One adult rat per box was picked randomly out of each box for this study. Five juvenile rats were collected randomly from 4 different boxes that contained the juvenile rats. When handling the rats, researchers used face masks (protective class FFP-2), disposable gloves, and coveralls. Serum and lung samples of the adults and juveniles were collected and analyzed as described previously.

The owner of the rat farm did not keep a record of where he bought and sold his rats. He reported that he regularly sold rats to several feeder rat breeding farms within the Netherlands. These farms were subsequently contacted, but their management would not cooperate with the investigation.

### Sequencing

From 1 rat owned by the patient and 1 rat from the breeding farm, the complete SEOV genome (i.e., the small, medium [M], and L segments) was sequenced. Primers were developed based on published sequences and are available on request. All fragments were purified with ExoSAP-IT PCR clean-up (Isogen Life Science) and sequenced by Baseclear.

## Results

### Patient and Close Contacts

Testing found high antibody titers to the orthohantaviruses, especially SEOV, in the patient. The patient improved in 4 days, platelet count and liver enzyme test improved, and he was discharged. A second serum sample for detection of antibodies to orthohantaviruses was taken 3 weeks later when the patient had made a full recovery.

The patient was found to be positive in the orthohantavirus IFA with the highest IgG and IgM titers to SEOV (Table 2). Orthohantavirus RNA was not detected in the serum sample. Two close contacts of the patient tested negative in serologic testing for orthohantaviruses. The first close contact was the father of the index patient. While the index patient was hospitalized, his father had fed several rats to the reptiles. The second close contact was the cohabiting partner of the index patient and reported no contact with the rats.

**Table 2:** Serologic orthohantavirus results for the patient with Seoul virus infection and 2 close contacts, the Netherlands, September 2016*

Sample	Age, y	SEOV IgG	SEOV IgM	DOBV IgG	DOBV IgM	SAAV IgG	SAAV IgM	HNTV IgG	HNTV IgM	PUUV IgG	PUUV IgM	SNV IgG	SNV IgM
Patient sample 1†	28	1:16,384	1:4,096	1:2,048	1:2,048	1:512	1:1,024	1:16,384	1:512	1:64	1:32	1:512	1:64
Patient sample 2‡		1:32,768	1:2,048	1:8,192	1:512	1:2,048	1:256	1:16,384	1:1,024	1:512	1:64	1:512	1:128
Close contact 1 (father)	59	–	–	–	–	–	–	–	–	–	–	–	–
Close contact 2 (partner)	27	–	–	–	–	–	–	–	–	–	–	–	–

*DOBV, Dobrava-Belgrade orthohantavirus; HNTV, Hantaan orthohantavirus; PUUV, Puumala orthohantavirus; SAAV, Saaremaa orthohantavirus; SEOV, Seoul orthohantavirus; SNV, Sin Nombre orthohantavirus; –, negative. †Taken 7 days after onset of symptoms. ‡Taken 31 days after onset of symptoms (reconvalescent).

### Feeder Rats from Patient

Of the 10 rats collected from the patient’s home, 6 (2/5 fresh and 4/5 frozen) rats were found positive by serologic testing and rRT-PCR (Table 3). All 5 fresh rats were tested in the nested rRT-PCR assay of the L segment, and again, the same 2 rats were positive.

**Table 3:** Results of Seoul virus tests in rats from the patient’s residence and the rat breeding farm, the Netherlands, September 2016

Source	Tested rats	No. (%) seropositive rats	No. (%) rats found to be positive by rRT-PCR
Feeder rats of the patient, n =10	5 fresh adults	2 (40)	2 (40)
	5 frozen adults	4 (80)	4 (80)
Feeder rats from the farm, n = 8,000–9,000	40 adults	40 (100)	40 (100)
	16 juveniles	1 (6)	0 (0)

*rRT-PCR, real-time reverse transcription PCR.

### Feeder Rats Breeding Farm

Of 60 rats purchased from the rat breeding farm, 4 juveniles died from poor condition before they could be euthanized. The remaining 40 adults and 16 juveniles were tested using ELISA and rRT-PCR. All 40 adult rats were seropositive for orthohantaviruses. Lung tissues of all adult rats tested positive for SEOV RNA by rRT-PCR. A selection of 5 adult rats was tested with the nested rRT-PCR assay of the L segment, and all 5 rats were positive.

Of the juveniles, 1 of the 16 was found to be seropositive. However, all 16 were orthohantavirus negative by rRT-PCR on lung tissues (Table 3).

### Sequencing

**Figure 2:**
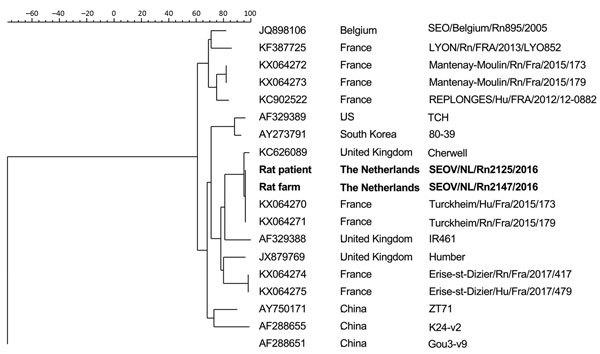
Phylogenetic tree calculated for the coding region (1,290 bp) of the small segment of the nucleocapsid protein in the Seoul virus strain implicated in a human case infection in the Netherlands, September 2016, compared with reference viruses. Boldface indicates isolates from this study; GenBank accession numbers are provided for reference viruses.

The 7 SEOV-positive rats (2 from the patient and 5 from the rat breeding farm) tested by nested rRT-PCR showed identical sequences of the L segment. The complete SEOV genome was sequenced from 1 of the patient’s rats and 1 breeding farm rat. Sequences were submitted to GenBank (accession nos. MG764078–83). The phylogenetic tree of the small segments (Figure 2) shows the strains are 100% identical. Furthermore, these strains are 100% identical to the Turckheim strain isolated from pet rats in France (*11*) and 99.6% identical to the Cherwell strain (*3*) isolated from pet rats in the United Kingdom. Also, the M segment were identical to each other and 99.8% identical to the Cherwell strain. The L segment was 99.6% identical to the Cherwell strain. The sequence of the M and L segments of the Turckheim strain were not available for comparison.

## Discussion 

SEOV was detected in wild brown rats in the Netherlands in 2013, but no human cases had been reported. In this article, we report an autochthonous case of SEOV infection in the Netherlands. The case-patient had nonspecific clinical symptoms of an orthohantavirus infection, showing gastrointestinal symptoms and distinct elevation of liver enzymes. Although the patient did not develop acute kidney injury, we found mild proteinuria and thrombocytopenia, which might also be found in SEOV infection. Because SEOV infections are related to a mild form of HFRS, with low incidence of hemorrhagic manifestations and low mortality rates, previous cases might have been missed or misdiagnosed as viral infection or gastroenteritis. Elevation of liver enzymes has been reported to occur to a greater extent in SEOV infections but in this patient might have been attributable to use of medication (acetaminophen) or gamma-hydroxubutyrate. Diagnosis of HFRS attributable to SEOV infection is largely based on serologic testing.

The patient had high SEOV IgG and IgM titers (1:16,384 for IgG and 1:4,096 for IgM) by IFA. Cross-reactions exist within 2 defined serogroups of orthohantaviruses but are limited between the serogroups. Antibodies against Puumala virus and Sin Nombre virus cross-react, whereas SEOV as member of the other serogroup cross-reacts with Hantaan virus, Dobrava-Belgrade virus, and Saaremaa virus. This investigation found a clear link from the patient to the SEOV-infected rats at home and at the breeder farm. Combined with the high antibody titers against SEOV, we concluded that the patient’s positive IFA result was due to a recent SEOV infection.

The patient’s rats probably originated from the breeding farm where the patient worked as a volunteer, which is also suggested by the sequence results. Management at the farm facilitated spread of SEOV by frequently moving rats from one box to the other and randomly returning female rats to boxes after they had weaned their pups. No registration of these movements was recorded. The tested rats were in a poor body condition, and many had bite wounds on their ears, providing ideal transmission conditions for SEOV (*19*). Several health risks (including public health risks) were identified with regard to the SEOV-positive feeder rat breeding farm, including the possibility of infection of personnel and visitors the farm and the potential spread of the pathogen by regular trade and exchange with other feeder rat breeding farms and occasionally with unknown parties, possibly including pet stores. Also, SEOV might spread from the feeder rats to wild rats living around the farm through direct contact or through contaminated materials (e.g., bedding material). Anyone entering the breeding farm was advised to wear disposable gloves, shoe sleeves, an apron, and a face mask (protective class FFP-2) and to wash their hands immediately after leaving the barn. They were also warned to avoid being bitten by the rats. All employees and volunteers were offered a blood test to see if they had been infected; no one accepted. Anyone buying or otherwise taking rats from the breeding farm was given a letter, signed by the Municipal Health Services, informing them of the possibility of the rats being infected. Pest control measures were set up, preventing contact of wild rats with feeder rats. The distribution of the used sawdust over nearby farm lands was discontinued. Used sawdust was thereafter brought to the municipal facility to be burnt in a waste disposal facility. By law in the Netherlands, notification of orthohantavirus infection in humans is mandatory but not in animals. Therefore, no legislation on control measures (e.g., enforced quarantine of infected animals, a ban on selling rats, or a forced closure of the breeding farm) was in effect. These limitations complicated source investigation because testing of the breeder rat farm was based on voluntary cooperation of the farm owner; they also severely complicated our efforts to contain this virus.

Duggan et al. showed that the seroprevalence of antibodies to SEOV in persons with a high contact rate with rats, such as rat owners, is 34%, compared with 3% in controls with occupational exposure to pet fancy rats or wild rats (*12*). Naturally, this probability depends on the spread of SEOV in the domestic rat populations in the Netherlands. To what extent SEOV is present in rat populations in the Netherlands is unknown. Hantavirus-infected rodents do not show any overt symptoms and might spread orthohantavirus for a prolonged period, possibly lifelong (*20*–*23*). However, the presence in feeder rats and anecdotal information about exchange between rat populations suggest that SEOV might be present in captive rat populations in the Netherlands. Also, exchange of pet and feeder rats between countries in Europe might be extensive, which is supported by the close resemblance of the SEOV strain in the Netherlands to the Cherwell strain in the United Kingdom and the Turckheim strain in France. In Europe, SEOV has been detected in pet rats in England and Wales (*13*), Sweden (*5*), and France (*11*).

This case illustrates the importance of clinical awareness for orthohantavirus infections after contact with rodents, including in patients with nonspecific symptoms, and the challenges that arise when source investigation and implementation of control measures are hampered by lack of legislation. The source investigation and implementation of control measures required multidisciplinary, constructive cooperation between research institutions and authorities. Future studies to assess the extent of SEOV infection in the domestic rat populations in the Netherlands are needed to inform the general public concerning the risk for contracting this virus by handling rats and the related health risks.
